# Repeatability and reliability of left ventricular focused cardiac ultrasound parameters in dogs obtained and measured by two non-cardiologist clinicians

**DOI:** 10.1093/jvimsj/aalaf090

**Published:** 2026-02-14

**Authors:** Christopher R Kennedy, Aurélie Jourdan, Kris Gommeren, Anne-Christine Merveille

**Affiliations:** Département clinique des animaux de compagnie, Université de Liège, Liège, Belgium; Veterinary Teaching Hospital, Clinical Veterinary Medicine, University of illinois at Urbana-Champaign, Urbana, IL, United States; Emergency and Critical Care, Centre Hospitalier Vétérinaire FREGIS, Paris, France; Département clinique des animaux de compagnie, Université de Liège, Liège, Belgium; Département clinique des animaux de compagnie, Université de Liège, Liège, Belgium

**Keywords:** echocardiography, focused cardiac ultrasound, left ventricle

## Abstract

**Background:**

Focused cardiac ultrasound (FCU) of the left ventricle (LV) is performed by non-cardiologists in critical care settings. Validity of measurements relies on accurate acquisition and measurement techniques.

**Hypothesis/Objectives:**

Investigate the acquisition and measurement repeatability of selected LV parameters and report reliability compared with measurements made by a board-certified cardiologist, with the goal of identifying parameters that might warrant further investigation in FCU.

**Animals:**

Thirty hemodynamically stable dogs with sinus cardiac rhythms.

**Methods:**

Right-sided FCU was performed by 2 critical care clinicians and a cardiologist. Left ventricular (LV) diameters, fractional shortening, and E-point septal separation (EPSS) were recorded. Intra-operator acquisition repeatability was quantified by coefficients of variation (CV_acquisition_); inter-operator reliability (compared with the cardiologist) was described by intraclass correlation coefficients (ICC_inter-operator_) and CV_inter-operator_; intra-operator measurement repeatability was described by ICC_measurement_.

**Results:**

Left ventricular end-diastolic diameter (LVEDD) in short-axis B-mode had excellent repeatability and reliability for both critical care clinicians (CV_acquisition_ < 10%; CV_inter-operator_ < 10%, ICC_inter-operator_ > 0.9; ICC_measurement_ > 0.9). Left ventricular end-diastolic diameter in short axis M-mode and long axis B- and M-mode showed good repeatability and reliability. E-point septal separation was neither repeatable nor reliable.

**Conclusions and clinical importance:**

The LVEDD measurements were repeatable and reliable when obtained and measured by 2 non-cardiologists in hemodynamically stable dogs with normal sinus rhythms. Further study of LVEDD measurements is recommended in FCU. The EPSS cannot be recommended based on these data.

## Introduction

Focused cardiac ultrasound (FCU) of the left ventricle (LV) has been investigated for evaluating dogs in respiratory distress and predicting fluid responsiveness (an increase in stroke volume after fluid administration).^1–3^ Although these studies support using LV FCU in dogs in emergency and critical care (ECC), questions regarding its repeatability and reliability when performed by non-cardiologists remain, and its role in assessing LV structure and function remains unclear.

Two ECC-relevant studies investigated LV short-axis M-mode echocardiography to predict fluid responsiveness.^2^^,^^3^ These studies normalized left ventricular end-diastolic diameter (LVEDD) to body weight to adjust for patient size differences.^4^ One study included 26 conscious, spontaneously breathing dogs and found that dogs with normalized LVEDD ≤ 1.34 responded to fluids.^2^ A second study identified a similar cut-off of < 1.32 in 44 spontaneously-breathing, hemodynamically-compromised dogs.^3^ Both studies used experienced sonographers. A third study showed that including FCU permitted non-cardiologists to accurately differentiate between cardiac and noncardiac causes of respiratory distress.^1^ Although using LV FCU seems promising, the reliability of novice sonographer measurements remains uncertain.

Four studies explored non-cardiologist FCU in non-ECC settings, evaluating LV linear dimensions, including LVEDD and left ventricular end-systolic diameter (LVESD), using M-mode or B-mode echocardiography.^5–8^ Importantly, LVEDD is a marker of preload, whereas LVESD is a marker of systolic function. Sonographers of variable experience had good agreement in LVEDD and LVESD measurements compared to 2 cardiologists.^7^ Another study using healthy beagles showed low variation in LVEDD and LVESD measurements between novice sonographers and a cardiologist.^5^ The latter study reported relatively higher variation in fractional shortening (FS), another marker of systolic function. E-point septal separation (EPSS), a different marker of systolic function^9^^,^^10^ used in FCU in humans, was not investigated.^11^^,^^12^ Unlike FS, EPSS requires only one measurement, theoretically minimizig measurement error, and making it an interesting parameter to investigate.

Two studies were slightly less supportive of LV parameters evaluated by non-cardiologists.^6^^,^^8^ A study evaluating trained non-cardiologists identifying stages of myxomatous mitral valve disease (MMVD) in dogs found they underestimated LVEDD. Although differences were small, they could lead to underdiagnosis of stage B2 disease.^6^ Our study included the natural caseload of non-cardiologist practitioners, rather than a controlled setting, which suggests its results might be more reflective of real-world scenarios. A similar study showed trained veterinary students also underestimated LVEDD.^8^ In the context of FCU for identifying cardiac disease or an underloaded LV to predict fluid responsiveness, even minor underestimations may be clinically relevant.

Although subjective assessment of LV size and function can be helpful in emergency patients^1^, identifying repeatable and reliable LV parameters, and the optimal imaging planes (short- vs long-axis) and modes (M-mode or B-mode), should allow for objective and comparable conclusions. We aimed to evaluate the repeatability and reliability of several LV FCU parameters measured by 2 non-cardiologists and compare them to measurements made by a cardiologist. The goal was to identify parameters that merit further investigation. We hypothesized that (1) ECC clinicians can repeatably obtain the FCU parameters, (2) their measurements are reliable when compared to a cardiologist, and (3) they can repeatably measure these parameters when given ideal images.

## Materials and methods

A prospective exploratory, single-center, observational study was performed in 3 parts to investigate (1) intra-operator acquisition repeatability, (2) inter-operator reliability, and (3) intra-operator measurement repeatability. For repeatability analyses, 2 repeated measurements were used. For reliability, parameters obtained and measured by an ECC intern and a criticalist were compared to those obtained and measured by a board-certified cardiologist. We used the following definitions, based on the American Society of Echocardiography^13^: repeatability is the “variation in repeat measurements made on the same subject under identical conditions” and reliability is the “magnitude of error between repeated measurements.” We defined variation as the dispersion of data, with more dispersion indicating more variation.

### Study design

Client-owned dogs hospitalized at the University of Liège between May 24th, 2022 and August 30th, 2022 were recruited. We aimed not to generalize findings to all dogs needing cardiac ultrasonography, but to identify repeatable and reliable parameters to investigate further. The target population was ECC clinicians, and it was assumed that the 2 ECC clinicians represented the broader ECC practitioner population. The target population in terms of species was all dogs that might be presented or admitted to a veterinary hospital. The source population included all hospitalized dogs—admitted through the emergency room, in the intensive care unit (ICU), or in lower-acuity wards—to provide a diverse range of morbidities and demographics typical of a general hospital population. Dogs were eligible for enrollment if they were hemodynamically stable with a sinus cardiac rhythm and tolerated restraint in right lateral recumbency. Dogs were excluded if (1) they became distressed during the examination, or (2) we were unable to obtain diagnostic quality (as assessed by the individual examiner in real-time) cineloops for all examiners for each patient. No dogs were sedated or received additional medications for the purpose of our study. Patient data collected included age (years), weight (kg), sex, breed, diagnosis, presence or absence of cardiac disease, and cardiac rhythm. Limited data were available upon which to base a sample size calculation. For our exploratory study, we estimated our sample size requirement based on a commonly used method for planning reliability studies using intraclass correlation coeffiecient (ICC).^14^ With an expected ICC (ρ₁) of 0.90, a minimally acceptable ICC (ρ₀) of 0.80, 3 sonographers, a significance level of 0.05, and statistical power set to 0.80, 18 dogs would have been needed, assuming each dog was only evaluated once by each sonographer. To further ensure adequate precision of the ICC estimate, we increased this number to 30 dogs, so that the lower bound of the 95% CI would remain above 0.80, as recommended previously.^15^ However, we emphasize that our study was exploratory, seeking to identify parameters that might warrant further evaluation. The results of our study may be used to base sample size calculations for future studies. Our study was approved by the Ethical Committee of the University of Liège. On admission to the hospital, all clients signed an informed consent form for using these data collected from their pets in research and teaching.

### The FCU examination

Three clinicians performed the FCU examinations using an ultrasound machine (DC-80A ultrasound, Mindray, China). One was an ECC intern (ECC#1) with FCU experience limited to clinical practice, 1 was a criticalist (ECC#2) with 7 discontinuous weeks of experience working in cardiology clinics, and the last was a board-certified cardiologist. Before the study, ECC#1 and ECC#2 underwent a 1-h hands-on training session with the cardiologist, where they were instructed on obtaining the appropriate views for the study, the use of B-mode and M-mode echocardiography and how to perform the measurements.

Cardiac ultrasonography was performed as previously described.^16^ Dogs were placed in right lateral recumbency on an echocardiography table. Electrocardiography leads were attached. The hair obscuring the ultrasound window was clipped and alcohol was applied. Phased array probes were used: a 3-7 MHz probe (P7-3, Mindray, China) was used for dogs < 15 kg and a 2-4 MHz probe (P4-2, Mindray, China) probe was used for dogs > 15 kg. Coupling gel was used to improve contact. Table 1 lists the studied parameters, the views by which they were obtained and how they were measured. Measurements were made offline on the ultrasound cart.

**Table 1:** Measurement of selected cardiac parameters.

Cardiac view	Placement of the probe	Cardiac parameters	Measurement
**Right parasternal long-axis 4-chamber view (PLAX)**	Probe on the right lateral chest wall where the apex beat is, parallel to the long axis of the heart, with the ultrasound marker pointed craniodorsally. Adjust to maximize left ventricular size. Ensure the leaflets of the mitral valve are visible throughout diastole and systole.	PLAX LVEDD-B PLAX LVESD-B	Using digital calipers, a line is drawn transecting the left ventricle at its widest diameter, perpendicular to the septum, avoiding the inflection at the base of the septum. Record a cine loop. Measure the internal diameter in diastole, timed to the peak of the Q-wave on the ECG, and in systole at the narrowest point after the QRS complex, using an inner-edge-to-inner-edge technique.
		PLAX LVEDD-M PLAX LVESD-M	Activate M-mode with the cursor after the same line as above for B-mode. Record a cine loop. Using digital calipers, the left ventricular diastolic diameter is measured timed to the peak of the ECG Q-wave and the systolic diameter is measured at the narrowest point after the QRS complex. A leading-edge-to-leading-edge technique is used.
		PLAX EPSS-B PLAX EPSS-M	In B-mode and M-mode, using digital calipers, the minimum distance between the interventricular septum and the tip of the mitral valve at the peak of the E-wave is measured.
		PLAX FS-B PLAX FS-M	Calculated (LVEDD—LVESD/LVEDD × 100)
**Right parasternal short-axis (PSAX) transventricular view**	From the long-axis view, rotate 90° counterclockwise. Slide and/or angle until a view of the papillary muscles (just below the mitral valve) appears.	PSAX LVEDD-B PSAX LVESD-B	Using digital calipers, a line is drawn transecting the left ventricle at its widest diameter, perpendicular to the septum, bisecting the ventricle into 2 halves. Record a cine loop. Measure the internal diameter in diastole timed to the peak of the Q-wave on the ECG and in systole at the narrowest point after the QRS complex, using an inner-edge-to-inner-edge technique.
		PSAX LVEDD-M PSAX LVESD-M	Activate M-mode with the cursor after the same line as above for B-mode. Record a cine loop. Using digital calipers, the left ventricular diastolic diameter is measured timed to the peak of the ECG Q-wave and the systolic diameter is measured at the narrowest point after the QRS complex. A leading-edge-to-leading-edge technique is used.
		PSAX FS-B PSAX FS-M	Calculated (LVEDD − LVESD/LVEDD × 100)
**Right parasternal short-axis (PSAX) transvalvular view**	From the short-axis view, tilt the probe craniodorsally until the leaflets of the mitral valve are seen.	PSAX EPSS-B PSAX EPSS-M	In B-mode and M-mode, using digital calipers, the minimum distance between the interventricular septum and the tip of the mitral valve at the peak of the E-wave is measured.

B or M suffixes denote B-mode and M-mode.

Abbreviations: EPSS = E-point septal separation; FS = fractional shortening; LVEDD = left ventricular end-diastolic diameter; LVESD = left ventricular end-systolic diameter.

Five dogs were included in part one (intra-operator acquisition repeatability analysis). All 3 clinicians performed the FCU examination and measurements on each of these 5 dogs twice, nonconsecutively, within 1 h. Thirty dogs were included in part two (inter-operator reliability analysis). All 3 clinicians performed the FCU examination and measurements on each of these dogs once, within 1 h. For part three (intra-operator measurement repeatability analysis), the cineloops of the 30 dogs obtained by the cardiologist were masked and reviewed twice with a 4-week period between reviews, in a randomized order, by the 2 ECC clinicians.

### Statistical analyses

For part one, intra-operator acquisition repeatability was evaluated using the coefficient of variation (CV_acquisition_), which provides a measure of variability.^17^ For part two (the reliability analysis), individual sets of data recorded by the 2 ECC clinicians were compared to data from the cardiologist using 2 different methods: (1) coefficients of variations (CV_inter-operator_) were calculated and Bland–Altman plots were generated using the cardiologist as the “gold standard” to which the ECC clinicians were compared^18^, and (2) the interclass correlation coefficient (ICC_inter-operator_) with its 95% CI. In the Bland–Altman plots, proportional bias was tested by calculating the correlation between the mean (x-axis) and the difference (y-axis) between the cardiologists' and the ECC clinicians’ measurements. The ICC measures correlation and agreement (ie, reliability), and provides a reliability index.^19^ For part three, the intra-operator measurement repeatability was assessed using ICC_measurement_ and 95% CI. In addition, a 2-way analysis of variance (ANOVA) model (generalized linear model, GLM) was used to test if there were any specific effects on the results obtained due to (1) the specific parameter that was being measured (round 1 or 2), or (2) the individual making the measurement. For the GLM, the outcome variable was each of the 16 parameters measured; the explanatory variables were the measurements themselves and the imagers making the measurements.

Results are reported as mean (SD), or median (minimum-to-maximum). Statistical test results were interpreted as follows. For CVs, mean values > 20% were considered representative of high dispersion of data and mean values < 10% represented low dispersion of data.^20^ For interpretation, CVs < 10% were considered “excellent,” 10 to ≤ 15% “good,” 15 to ≤ 20% “fair,” and > 20% “poor” variation. The ICC was interpreted based on the lower limits of the 95% CI: < 0.5, 0.5-0.75, 0.75-0.9, and > 0.9 were representative of poor, moderate, good, and excellent reliability, respectively.^19^ For interpretation of the GLM, *P*-values < .05 were considered significant. Statistical analyses were performed using SAS version 9.4 (SAS Institute, Cary, NC, USA) and figures were constructed in R version 4.2.2.

## Results

### Intra-operator acquisition repeatability

Five healthy dogs were enrolled, including 2 Border Collies, 1 Golden Retriever, 1 Maltese Terrier, and 1 mixed breed dog. The median age and median weight were 4 years (minimum-maximum: 1-9 years) and 16.5 kg (minimum-maximum: 8-25 kg). No dogs had cardiac disease. Table 2 shows the CV_acquisition_ for the selected parameters. Parameters with < 10% CV_acquisition_ (ie, low or “excellent” variation) for all clinicians included parasternal long-axis (PLAX) LVESD-B, PLAX LVEDD-M, PLAX LVESD-M, parasternal short-axis (PSAX) LVEDD-B, and PSAX LVEDD-M. For the linear dimensions, both ECC clinicians had generally good or excellent variation, similar to the cardiologist, indicating that these parameters were repeatably acquired and measured by the same sonographer twice. The exception was PSAX LVESD-B for ECC#1, which had fair variation (CV 15.4%). Although FS had variations ranging from excellent to fair for the cardiologist and ECC#2, for ECC#1 only PSAX FS-M had fair variation, with the other FS parameters having poor variation, indicating that they were not repeatable for this clinician. For both ECC clinicians, like the cardiologist, the EPSS measured in B-mode showed poor variation, indicating that, under identical conditions, different values were obtained. M-mode EPSS had good variation for both the cardiologist and ECC#2 measured in long-axis, though fair variation for ECC#1; in short-axis, M-mode EPSS showed fair variation for the cardiologist and ECC#2, and poor variation for ECC#1.

**Table 2:** Intra-operator acquisition repeatability.

	Cardiologist	ECC#1	ECC#2
**PLAX LVEDD-B**	**2.73** (0.33-0.51)	10.4 (1.27-19.4)	**2.98** (0.36-5.6)
**PLAX LVESD-B**	**6.07** (0.74-11.4)	**7.16** (0.87-13.4)	**6.10** (0.74-11.5)
**PLAX FS-B**	14.1 (1.73-26.6)	*36.5* (4.45-68.6)	18.6 (2.27-35.0)
**PLAX LVEDD-M**	**1.34** (0.16-2.51)	**1.74** (0.21-3.27)	**3.02** (0.37-5.67)
**PLAX LVESD-M**	**3.40** (0.41-6.39)	**6.56** (0.8-12.32)	**5.45** (0.67-10.24)
**PLAX FS-M**	11.1 (1.35-20.8)	*21.3* (2.60-40.1)	**9.06** (1.10-17.01)
**PLAX EPSS-B**	*26.2* (3.19-49.1)	*74.9* (9.13-140.6)	*33.5* (4.09-62.9)
**PLAX EPSS-M**	13.0 (1.59-24.4)	17.7 (2.15-33.2)	11.4 (1.39-21.3)
**PSAX LVEDD-B**	**2.87** (0.35-5.39)	**7.30** (0.89-13.7)	**3.85** (0.47-7.23)
**PSAX LVESD-B**	**5.13** (0.63-9.63)	15.4 (1.88-28.9)	**5.21** (0.64-9.78)
**PSAX FS-B**	11.7 (1.43-22.0)	*46.5* (5.67-87.3)	10.7 (1.31-20.2)
**PSAX LVEDD-M**	**4.67** (0.57-8.78)	**6.16** (0.75-11.6)	**4.55** (0.56-8.55)
**PSAX LVESD-M**	12.2 (1.49-22.9)	**9.33** (1.14-17.5)	**4.54** (0.55-8.52)
**PSAX FS-M**	16.2 (1.98-30.5)	18.6 (2.26-34.9)	**4.54** (0.55-8.52)
**PSAX EPSS-B**	*23.7* (2.89-44.5)	*36.3* (4.42-68.1)	*20.3* (2.47-38.0)
**PSAX EPSS-M**	19.2 (2.34-35.97)	*30.7* (3.75-57.7)	16.7 (2.04-31.3)

Coefficient of variation (CV) percentages (mean and 95% CIs) between the first round of data collection and the second round for individual clinicians. Mean CV < 10% are highlighted in bold, mean CV > 20% are highlighted in italics. Number of dogs (*n*) = 5.

B or M suffixes denote B-mode and M-mode.

Abbreviations: ECC = emergency and critical care; EPSS = E-point septal separation; FS = fractional shortening; LVEDD = left ventricular end-diastolic diameter; LVESD = left ventricular end-systolic diameter; PLAX = parasternal long-axis; PSAX = parasternal short-axis.

### Inter-operator reliability

Breeds included mixed breed dogs (*n* = 5), Border Collies (*n* = 4), Golden Retrievers (*n* = 3), Australian Shepherd dogs (*n* = 2), French Bulldogs (*n* = 2), and 1 each of Beauceron, Boxer, Chihuahua, Cavalier King Charles, Cocker Spaniel, Dachshund, Dalmatian, Doberman Pinscher, Labrador Retriever, Maltese, Polish Shepherd Dog, Springer Spaniel, Shi Tzu, and West Highland White Terrier. The median age was 5.5 years (minimum–maximum: 0.5-12 years) and the median weight was 14.5 kg (minimum–maximum: 2.4-47 kg). Eleven dogs were spayed females, 8 dogs were intact males, 7 were neutered males, and 4 were intact females. All dogs were hemodynamically stable with cardiac sinus rhythms at the time of the echocardiography (28/30 had normal sinus rhythm, 2/30 had sinus arrhythmia). The primary disease processes included: healthy (*n* = 5), septic peritonitis (*n* = 3), spinal myelopathy (*n* = 4), and 1 each of hypoadrenocorticism, acute kidney injury, brachycephalic airway syndrome, cholangiopathy, endocarditis, nonspecific fever, gastroenteritis, gastrotomy, immune-mediated hemolytic anemia, laryngeal mass, mass removal, palpebral mass, portosystemic shunt, pyothorax, right hindlimb amputation, seizures, severe soft tissue infection, and generalized tetanus. Five dogs had cardiac disease (or diseases closely related to the heart): 1 had mitral endocarditis, 1 had MMVD stage B1, 1 had mild pulmonary hypertension, 1 had mild systolic dysfunction, and 1 had a scant amount of incidental pericardial effusion.

**Figure 1:**
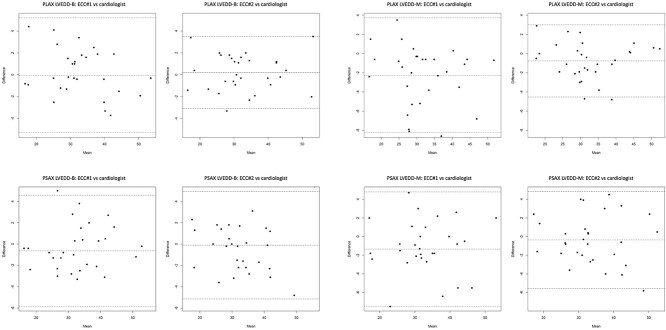
Bland–Altman plots for the left ventricular end-diastolic diameters (LVEDDs) in B-mode (-B) and M-mode (-M) in long-axis (PLAX) and short-axis (PSAX) for each ECC clinician vs the cardiologist. The x-axis represents the mean of the measurements made by the specific ECC clinician and the cardiologist; the y-axis represents the difference between the measurements made by the specific ECC clinician and the cardiologist. Units are in millimeters (mm). The middle, dashed line depicts the mean difference. The top and bottom dashed lines depict the 95% limits of agreement.

Table3 shows the CV_inter-operator_ and the ICC_inter-operator_ comparing the ECC clinicians to the cardiologist. Parasternal long-axis LVEDD-B, PLAX LVEDD-M, PSAX LVEDD-B, PSAX LVEDD-M, and PSAX LVESD-M had < 10% CV_inter-operator_ for both the ECC#1 and ECC#2, indicating that these measurements varied little from the measurements by the cardiologist. For ECC#2 but not ECC#1, PLAX LVESD-B and PSAX LVESD-B also had < 10% CV_inter-operator_; for the ECC#1, both of these had good variation (CV_inter-operator_ 10.7% and 12.2%, respectively). Parasternal long-axis LVEDD-B and PSAX LVEDD-B had excellent reliability (ICC_inter-operator_ and 95% CI > 0.9) for both ECC#1 and ECC#2, indicating that these measurements were in excellent agreement with those made by the cardiologist. Parasternal long-axis LVESD-B, PLAX LVEDD-M, PSAX LVESD-B, and PSAX LVEDD-M also had excellent reliability for ECC#2. Figure 1 shows the Bland–Altman plots for the LVEDD parameters.

**Table 3:** Comparisons between the ECC clinicians and the cardiologist (*n* = 30 dogs).

Parameter	Clinician	Adjusted difference	Correlation between mean vs difference	ICC	CV (%)
		(Mean ± SD)	*r* (*P*-value)		
**HR, bpm**	ECC#1	−0.83 ± 16.8	−0.11 (0.55)	0.897 (0.838-0.949)	11.6 (8.53-14.6)
	ECC#2	4.67 ± 12.0	0.091 (0.63)	**0.944 (0.904-0.974)**	**8.69** (6.39-10.98)
					
**PLAX LVEDD-B**	ECC#1	−0.040 ± 2.68	−0.11 (0.58)	**0.958 (0.935-0.980)**	**5.73** (4.21-7.24)
	ECC#2	0.22 ± 1.68	0.023 (0.91)	**0.984 (0.975-0.992)**	**3.61** (2.66-4.56)
					
**PLAX LVESD-B**	ECC#1	0.98 ± 3.53	−0.18 (0.33)	0.884 (0.814-0.943)	10.7 (7.84-13.5)
	ECC#2	0.41 ± 2.23	0.20 (0.29)	**0.961 (0.938-0.981)**	**6.69** (4.92-8.45)
					
**PLAX FS-B**	ECC#1	−3.75 ± 11.1	0.14 (0.46)	*0.110 (−0.305-0.433)*	*30.6* (22.5-38.7)
	ECC#2	−0.46 ± 5.82	0.17 (0.38)	0.753 (0.613-0.874)	14.4 (10.6-18.2)
					
**PLAX LVEDD-M**	ECC#1	−2.26 ± 3.06	−0.15 (0.44)	0.909 (0.734-0.965)	**8.38** (6.17-10.6)
	ECC#2	−0.77 ± 1.92	−0.053 (0.78)	**0.974 (0.954-0.988)**	**4.43** (3.26-5.60)
					
**PLAX LVESD-M**	ECC#1	−1.25 ± 3.45	−0.045 (0.81)	0.889 (0.815-0.946)	11.7 (8.62-14.8)
	ECC#2	−1.91 ± 2.71	−0.33 (0.073)	0.901 (0.735-0.961)	10.8 (7.95-13.7)
					
**PLAX FS-M**	ECC#1	−0.87 ± 9.12	−0.002 ± (0.99)	0.569 (0.326-0.769)	19.6 (14.45-24.8)
	ECC#2	3.91 ± 6.75	−0.46 (0.010)	0.619 (0.329-0.810)	15.6 (11.5-19.8)
					
**PLAX EPSS-B**	ECC#1	−0.080 ± 1.41	−0.20 (0.29)	0.696 (0.524-0.843)	*33.2* (24.4-41.9)
	ECC#2	−0.040 ± 1.36	0.052 (0.78)	0.764 (0.630-0.880)	*31.6* (23.3-40.0)
					
**PLAX EPSS-M**	ECC#1	0.15 ± 0.84	0.28 (0.13)	0.817 (0.713-0.908)	*27.3* (20.1-34.5)
	ECC#2	−0.020 ± 1.14	0.47 (0.0083)	0.734 (0.583-0.864)	*37.9* (27.9-47.9)
					
**PSAX LVEDD-B**	ECC#1	−0.63 ± 2.66	−0.072 (0.71)	**0.958 (0.933-0.980)**	**5.7** (4.19-7.20)
	ECC#2	−0.11 ± 2.57	0.19 (0.32)	**0.965 (0.945-0.983)**	**5.32** (3.92-6.72)
					
**PSAX LVESD-B**	ECC#1	−2.54 ± 3.04	0.32 (0.087)	0.890 (0.619-0.960)	12.2 (8.96-15.4)
	ECC#2	−1.24 ± 2.05	0.35 (0.058)	**0.957 (0.900-0.982)**	7.14 (5.25-9.02)
					
**PSAX FS-B**	ECC#1	7.51 ± 10.0	0.68 (<0.0001)	0.453 (0.059-0.715)	*26.7* (19.7-33.8)
	ECC#2	3.97 ± 7.04	0.56 (0.0014)	0.644 (0.371-0.824)	18.2 (13.4-23.0)
					
**PSAX LVEDD-M**	ECC#1	−1.35 ± 3.13	−0.12 (0.52)	0.931 (0.877-0.968)	**7.15** (5.26-9.03)
	ECC#2	−0.36 ± 2.65	−0.11 (0.58)	**0.958 (0.933-0.980)**	**5.51** (4.06-6.97)
					
**PSAX LVESD-M**	ECC#1	−1.16 ± 2.12	0.028 (0.88)	0.950 (0.896-0.979)	**7.51** (5.52-9.49)
	ECC#2	−2.07 ± 2.05	0.17 (0.37)	0.932 (0.616-0.979)	**9.27** (6.82-11.7)
					
**PSAX FS-M**	ECC#1	1.18 ± 6.68	0.44 (0.015)	0.590 (0.362-0.781)	14.1 (10.4-17.9)
	ECC#2	5.97 ± 7.01	0.68 (<.0001)	0.531 (0.067-0.781)	18.0 (13.3-22.8)
					
**PSAX EPSS-B**	ECC#1	−0.13 ± 2.19	−0.075 (0.69)	0.577 (0.337-0.774)	*46.7* (34.4-59.0)
	ECC#2	−0.27 ± 1.02	−0.54 (0.0021)	0.886 (0.819-0.944)	*23* (17.0-29.1)
					
**PSAX EPSS-M**	ECC#1	−0.030 ± 0.82	0.13 (0.48)	0.901 (0.845-0.952)	*22.4* (16.5-28.4)
	ECC#2	−0.37 ± 0.86	0.18 (0.35)	0.875 (0.782-0.941)	*27.7* (20.41-35.1)

Difference is expressed in mm for all values except FS, where it is %. For ICC, parameters with the 95% CI > 0.90 are highlighted in bold and parameters with CI < 0.5 are highlighted in italics. For CV, parameters with mean CV < 10% are highlighted in bold and parameters with mean CV > 20% are highlighted in italics. Column 4 evaluates the correlation between the mean vs the difference, as depicted in the Bland–Altman plots (Figure 1): when *P* = < .05, proportional bias is present when left ventricular diameters are increasing (positive *r*) or decreasing (negative *r*).

B or M suffixes denote B-mode and M-mode.

Abbreviations: CV = coefficient of variation; ECC = emergency and critical care; EPSS = E-point septal separation; FS = fractional shortening; ICC = intraclass correlation coefficient; LVEDD = left ventricular end-diastolic diameter; LVESD = left ventricular end-systolic diameter; PLAX = parasternal long-axis; PSAX = parasternal short-axis.

The fractional shortening parameters showed higher variation and worse reliability compared with measurements obtained by the cardiologist, indicating that these measurements obtained by the ECC clinicians were not the same. Every EPSS parameter measured by both ECC clinicians showed poor variation, indicating that these values diverged from those reported by the cardiologist.

### Intra-operator measurement repeatability

Twenty-nine dogs were included in this part of the study. The patient with mitral endocarditis was excluded, because it had such obvious pathology that we were concerned examiners would recognize the case, despite the 4-week lapse time. The 4 other dogs with mild cardiac-related diseases were included.

Table 4 shows the ICC_measurement_ for each parameter for each ECC clinician and the GLM results. Parasternal long-axis LVEDD-B, PLAX LVEDD-M, PSAX LVEDD-B, PSAX LVESD-B, PSAX LVEDD-M, and PSAX LVESD-M had excellent reliability (ICC_measurement_ and 95% CI > 0.9) for both ECC#1 and ECC#2, indicating that, when presented with the same images twice, the ECC clinicians repeatably made the same measurements. Parasternal long-axis LVESD-B, PLAX LVESD-M, PLAX EPSS-B, and PSAX EPSS-B also had excellent reliability for ECC#2.

**Table 4:** ICC for each parameter for each ECC sonographer (*n* = 29 dogs).

Parameter	Clinician	Adjusted difference	ICC	GLM	Sonographer (*P*-value)
		Round 1 vs round 2 (mean ± SD)		Parameter (*P*-value)	
**PLAX LVEDD-B**	ECC#1	−0.22 ± 1.64	**0.984 (0.974-0.992)**	.39	.93
	ECC#2	−0.18 ± 0.97	**0.995 (0.992-0.998)**		
					
**PLAX LVESD-B**	ECC#1	−0.05 ± 3.75	0.882 (0.813-0.943)	.42	**<.0001**
	ECC#2	0.70 ± 1.68	**0.974 (0.953-0.988)**		
					
**PLAX FS-B**	ECC#1	−0.01 ± 0.12	*0.018 (-0.545-0.382)*	.19	**<.0001**
	ECC#2	−0.02 ± 0.05	0.786 (0.632-0.897)		
					
**PLAX LVEDD-M**	ECC#1	−0.66 ± 2.52	**0.962 (0.939-0.982)**	.11	.74
	ECC#2	−0.25 ± 1.07	**0.993 (0.989-0.997)**		
					
**PLAX LVESD-M**	ECC#1	−1.16 ± 1.75	0.960 (0.894-0.985)	.25	**<.0001**
	ECC#2	0.51 ± 1.84	**0.968 (0.948-0.985)**		
					
**PLAX FS-M**	ECC#1	0.03 ± 0.08	0.445 (0.146-0.690)	.63	**<.0001**
	ECC#2	−0.02 ± 0.06	0.798 (0.676-0.899)		
					
**PLAX EPSS-B**	ECC#1	0.30 ± 1.34	0.618 (0.401-0.799)	.29	.66
	ECC#2	0.10 ± 0.84	**0.939 (0.903-0.971)**		
					
**PLAX EPSS-M**	ECC#1	−0.20 ± 0.80	0.667 (0.476-0.828)	.83	**<.0001**
	ECC#2	0.25 ± 0.77	0.796 (0.670-0.899)		
					
**PSAX LVEDD-B**	ECC#1	0.37 ± 2.79	**0.954 (0.928-0.978)**	.42	**<.0001**
	ECC#2	0.07 ± 0.81	**0.997 (0.995-0.998)**		
					
**PSAX LVESD-B**	ECC#1	−0.37 ± 2.63	**0.945 (0.913-0.974)**	.86	.71
	ECC#2	0.47 ± 0.81	**0.993 (0.985-0.997)**		
					
**PSAX FS-B**	ECC#1	0.02 ± 0.08	0.569 (0.326-0.770)	.7	**.0024**
	ECC#2	−0.01 ± 0.03	0.902 (0.828-0.954)		
					
**PSAX LVEDD-M**	ECC#1	−0.39 ± 1.26	**0.990 (0.984-0.995)**	.36	.59
	ECC#2	−0.03 ± 0.86	**0.996 (0.994-0.998)**		
					
**PSAX LVESD-M**	ECC#1	−0.27 ± 2.08	**0.961 (0.939-0.982)**	.88	**<.0001**
	ECC#2	0.19 ± 1.64	**0.978 (0.965-0.989)**		
					
**PSAX FS-M**	ECC#1	−0.00 ± 0.06	0.632 (0.418-0.809)	.75	**<.0001**
	ECC#2	−0.00 ± 0.05	0.860 (0.778-0.932)		
					
**PSAX EPSS-B**	ECC#1	0.37 ± 1.26	0.622 (0.406-0.802)	.55	.2
	ECC#2	−0.16 ± 0.70	**0.951 (0.922-0.977)**		
					
**PSAX EPSS-M**	ECC#1	−0.17 ± 1.01	0.799 (0.683-0.900)	.46	**<.0001**
	ECC#2	0.34 ± 0.52	0.941 (0.851-0.976)		

Difference between repeated measurements *intra*-sonographer (ie, measurements made by the same sonographer) is measured in mm for all values except FS, where it is %. This analysis was performed using a GLM (a 2-way analysis of variance) with sonographer and measurement round as covariates. For ICC, parameters with the 95% CI > 0.90 are highlighted in bold and parameters with CI < 0.5 are highlighted in italics. The generalized linear model (GLM) significant values are highlighted in bold (*P* < .05).

B or M suffixes denote B-mode and M-mode.

Abbreviations: ECC = emergency and critical care; EPSS = E-point septal separation; FS = fractional shortening; ICC = intraclass correlation coefficient; LVEDD = left ventricular end-diastolic diameter; LVESD = left ventricular end-systolic diameter; PLAX = parasternal long-axis; PSAX = parasternal short-axis.

Moderate-to-poor measurement reliability was found for both ECC clinicians for PLAX FS-B, PLAX FS-M, and PLAX EPSS-M, indicating that these parameters were not measured the same twice. The GLM showed that there was a statistical difference in the repeated measurements obtained when the ECC clinicians performing the measurements were included as an explanatory variable. These differences indicated that the clinicians showed different aptitudes for making the same measurement twice. However, for PLAX LVESD-B, PLAX LVESD-M, PSAX LVEDD-B, and PSAX LVESD-M, whereas the GLM indicated a significant effect on the measurements obtained by different ECC clinicians, the ICC_measurement_ values showed good-to-excellent reliability for both clinicians repeatedly measuring these parameters.

## Discussion

This FCU study investigated 16 parameters of LV morphology and function, measured by different clinicians, in hemodynamically stable, hospitalized dogs with sinus rhythms. We studied intra-operator acquisition repeatability, inter-operator reliability (comparing ECC clinicians to a cardiologist), and intra-operator measurement repeatability. The parameter found to be most repeatable and reliable, considering all 3 parts of the study, was PSAX LVEDD-B. Other parameters that showed high degrees of repeatability and reliability were PLAX LVEDD-B, PLAX LVEDD-M, and PSAX LVEDD-M; all of these parameters are indicators of preload. These parameters warrant further investigation in FCU of dogs.

The repeatability and reliability of LV parameters are important. When measuring the same parameter twice, the variation must be low; if the variation in measurement of an unchanged parameter is higher than the degree of change that indicates a response (such as to a fluid challenge) that parameter cannot reliably indicate that response. A study investigating PSAX LVEDD-M as a predictor of fluid responsiveness found an average absolute change in LVEDD-M of 20% occurred in dogs that responded positively to fluid administration.^2^ That study also reported high repeatability of LVEDD measurements performed at baseline (ie, repeated measurements without fluid administration). Given the low variation observed in our study with LVEDD measurements performed by less experienced operators, it seems justified to study LVEDD measurements in critically ill dogs to determine if similarly low variation can be achieved and if these measurements may serve as reliable predictors and indicators of response to fluid administration.

Limited data are available for PSAX LVEDD-B, because focus has been given to PSAX LVEDD-M.^2^^,^^3^^,^^7^ Measuring LVEDD in B-mode is advantageous, because 2-dimensional images of the ventricle allow for accurate post-acquisition measurements bisecting the ventricle, whereas M-mode requires precise alignment through the ventricle’s center at the time of acquisition. In 12 dogs with cineloops recorded by a cardiologist and measurements made by a trained non-cardiologist, intra-operator measurement reliability was excellent (ICC [95% CI] = 0.99 [0.97-1.0]) for PSAX LVEDD-B.^21^ That study found excellent inter-operator reliability with measurements made by the trained measurer, a cardiologist, and a cardiology resident (ICC [95% CI] = 0.97 [0.94-0.99]). Similarly, in our study, PSAX LVEDD-B showed low intra-operator variation and high reliability. For LVEDD-B, in PSAX and PLAX, mean biases were close to zero for both clinicians compared with the cardiologist (Figure 1). These data suggest LVEDD-B may be useful to further study FCU. However, a study investigating 58 veterinary students showed LVEDD-B tended to be underestimated in dogs screened for MMVD.^8^ In our study, considering the limits of agreement, differences of 4 mm might occur. A 4 mm error must be considered in the context of ventricular size: for a 40 mm ventricle, a 10% error may not be unreasonable for less experienced sonographers; for smaller ventricles, this error is proportionately higher. This observation emphasizes the need for LVEDD-B to be studied in ECC settings in dogs, performed by non-cardiologists, on a sufficient scale to confirm margins of error and systematic biases, which would require multiple non-cardiologists, ideally at multiple centers, and a patient sample representative of those undergoing FCU in critical care settings, using dogs of different sizes.

Parasternal short-axis LVEDD-M is the most studied parameter. When measured by 2 cardiologists using healthy Boxers, the CVs for intra- and inter-operator variation were 8.19% and 7.69%.^22^ In 15 healthy beagles, measurements by 2 non-cardiologists compared to a cardiologist had CVs of 10.7% and 13.8% (“good” variation accordingly to our interpretation).^5^ In a study of 51 clinicians with variable echocardiography experience, PSAX LVEDD-M was moderately reliable (ICC = 0.73).^7^ A group of general practitioners trained to perform echocardiography for MMVD also had moderate reliability (ICC = 0.789) compared to cardiologists.^6^ In these 3 FCU studies, LVEDD-M was underestimated compared with measurements by their cardiologists. In our study, intra-observer variation for PSAX LVEDD-M was low, and measurement reliability was high. Compared to the cardiologist, PSAX LVEDD-M had low variability for both clinicians, excellent reliability for ECC#2 and good reliability for ECC#1. The LVEDD-M in PSAX and PLAX was mildly underestimated by ECC#1 (approximately −2 mm bias) and less so by ECC#2 (approximately < 1 mm bias; Figure 1). Although small underestimations may not be clinically relevant, underestimation and overestimation have resulted in disease misclassification by non-cardiologists for staging MMVD and may be clinically relevant in FCU performed in dogs.^6^^,^^7^

Parasternal long-axis LVEDD-B was reliable in our study in terms of consistency with the cardiologist and measurement repeatability. For ECC#1, the intra-operator acquisition variability displayed “good” variation (10.4%). A study of 12 dogs with cineloops obtained by a cardiologist reported excellent intra-operator reliability (ICC [95% CI] = 0.97 [0.92-0.99]) and inter-operator reliability (ICC [95% CI] = 0.98 [0.94-0.99]).^21^ In a different study, inter-operator reliability was excellent (ICC = 0.98) for 3 experienced echocardiographers.^23^ Given the similar results in our study, PLAX LVEDD may be a useful parameter in FCU.

Parasternal long-axis LVEDD-M showed low intra-operator variation in our study and the measurements were repeatable. For ECC#1, the reliability was moderate (ICC [95% CI] = 0.909 [0.734-0.965]). This mesurement has not been studied previously in FCU in dogs. Performed by cardiologists, the CVs for intra- and inter-operator variation for PLAX LVEDD-M were < 10%.^24^ Parasternal long-axis LVEDD-M might be a promising parameter to explore further; however, until more data are available, caution is recommended before using this parameter clinically.

In our study, the mean biases for LVEDD measurements vs those of the cardiologist were notably small, particularly for ECC#2. However, the limits of agreement were relatively large (Figure 1). A 4 mm difference can be clinically relevant, potentially leading to misclassification, for example, misclassifying stage B2 MMVD.^2^^,^^3^^,^^6^ Proportional bias, a systematic error that varies with the parameter’s value^13^, was identified for several parameters (Table 3). Although our exploratory study’s limited sample size prevents drawing firm conclusions, the data suggest the existence of proportional bias, warranting further investigation. This possibility is important because it relates to both the dog’s size and ventricular volume status, 2 variables not adequately addressed in our study.

In human medicine, LVEDD does not seem to be used regularly in the emergency or intensive care settings.^25^^,^^26^ Studies focus on subjective visual assessment of systolic function and EPSS.^27^ A study in emergency medicine did include LVEDD and LVESD to calculate FS but provided no more information on these linear measurements.^28^ In that study, FS was evaluated as a surrogate for LV ejection fraction (LVEF), highlighting the emphasis on LV systolic function that seems paramount in FCU in humans.^26^^,^^29^^,^^30^

We investigated 3 markers of systolic function in our study. Left ventricular end-systolic volume had promising results in terms of being repeatable and reliable. Results for FS and EPSS were less promising: FS was not reliable and generally had poor acquisition and measurement repeatability, particularly by ECC#1; EPSS was neither repeatable nor reliable. Left ventricular end-systolic volume, as a surrogate for end-systolic volume, is a marker of systolic function and is dependent on contractility and ventricular loading conditions.^9^ In our study, LVESD acquisitions were generally repeatable; compared to the cardiologist, reliabilty was reasonable; and measurement repeatability was good-to-excellent. Specifically, for ECC#2, PLAX LVESD-B and PSAX LVESD-B showed excellent reliability and low variation compared to the cardiologist, but reliability was relatively lower for ECC#1. A slight tendency to underestimate LVESD was observed for both ECC clinicians (Supplementary Material). These results are similar to a previous study that found “excellent” variation (CV 7.8%) and “good” variation (12.3%) for their 2 novice sonographers compared to a cardiologist, using PSAX LVESD-M.^5^ When measured by multiple sonographers with various levels of experience, PSAX LVESD-M showed good intra- and inter-operator reliability and moderate-to-good concordance with cardiologists.^7^ Left ventricular end-systolic volume assessments may be the most promising FCU tool for assessing systolic function. This situation is somewhat different to formal echocardiography by a cardiologist, where alternative measurements may be utilized because of the limitations of LVESD.^9^ Our results perhaps favor B-mode assessments, neither of which have been reported previously for FCU in dogs.

Parameters appearing least favorable for further study, based on highest variation and poorest reliability, were PLAX EPSS-B and PSAX EPSS-B; these parameters showed high intra- and inter-operator variation. Parasternal long-axis EPSS-M and PSAX EPSS-M showed high inter-operator variation. These results imply EPSS variables are neither repeatable nor reliable and might not warrant further exploration in FCU. To date, EPSS has not been studied in veterinary FCU. Because it only requires a single measurement, the risk of measurement error is theoretically less than FS, which requires 2 measurements plus an equation (ie, potential mismeasurement and miscalculation). However, the poor repeatability and reliability found in our study strongly caution against using EPSS. In human emergency medicine, EPSS had poor accuracy for identifying LVEF: with severely decreased LVEF, sensitivity was extremely low (0.11-0.18), but specificity was high (0.98-1.0); inter-operator reliability was moderate.^28^ Another FCU study in humans showed poor inter-operator reliability.^31^ When performed by 2 veterinary cardiologists, intra- and inter-operator CVs were poor (>20%)^19^, similar to our results. Combined, these data suggest that EPSS may be of limited value in veterinary FCU.

Fractional shortening showed high intra-operator and inter-operator variability in our study. This finding is interesting, because individually, the LV linear dimensions (LVEDD and LVESD) were promising, with generally good or higher repeatabilities and reliabilities. We believe the higher variability and lower reliability was caused by FS being a compound variable, where the small but present errors of both linear dimensions were compounded in the FS calculation. In human emergency medicine, FS was poorly accurate for identifying LVEF and showed poor inter-operator reliability.^27^ When performed by 2 veterinary cardiologists, intra- and inter-operator CVs were approximately 17.5%, and the authors cautioned against overinterpretation of changes < 18%.^22^ In our study, the CVs for FS parameters ranged from excellent to poor. Specifically, ECC#1 demonstrated fair variation for PSAX FS-M and poor variation for the other FS parameters. This observation indicates that FS parameters acquired and measured by ECC#1 were not repeatable and suggests that similar non-repeatability might occur with other non-cardiologists. The inter-operator reliability for all FS measurements by both ECC clinicians was mostly poor and the variation was mostly fair or worse. Combined, these data warrant caution if using FS in FCU, because accurate measurements and identifying true changes between 2 time points might not be possible.

Our study was not specifically designed to investigate training. However, a clear difference existed between ECC#1, who had one hour of hands-on training and prior experience limited to on-the-floor case management, and ECC#2, who had 7 weeks of experience working in cardiology clinics. The difference is seen in the GLM for measurement repeatability: the clinician performing the measurement affected several results. It is also inferable from the first and second parts of our study, where ECC#2 overall had superior acquisition repeatability and better reliability with measurements made by the cardiologist. In a veterinary FCU study, clinicians with hands-on experience achieved more accurate results than clinicians with predominantly theoretical knowledge.^7^ The effect of training programs should be investigated in veterinary FCU. Ahead of veterinary medicine, the European Society of Intensive Care Medicine offers the European Diploma in Advanced Critical Care Echocardiography program, which is a rigorous curriculum of clinical training, case reporting and conferences, plus 2 written examinations. Until such training is established in veterinary medicine, individuals performing FCU should critically consider their own proficiency when obtaining and reporting measurements and comparing between sequential measurements.

Limitations are inherent to FCU studies, with several important limitations in our study. The low number of dogs and clinicians limits the extrapolation of our data to the total population of dogs undergoing FCU by non-cardiologists. The data presented here may be used to guide sample size calculations for future studies. In addition, there are clear subpopulations, for example, patients in respiratory distress where FCU might be employed to investigate cardiac (hypervolemic) vs noncardiac causes^1^, and those in hemodynamic collapse where FCU might be employed to evaluate for hypovolemia.^2^^,^^3^ Our cases were hemodynamically stable, in sinus rhythms, and data might not extrapolate to unstable or arrhythmic patients. This limitation is difficult to avoid in FCU studies, and studying hemodynamically unstable patients might result in patient-related differences among observers or time points. In addition, heart rates were not identical within each dog for each clinician, nor did we time measurements with the respiratory cycle, both of which can introduce variation. We did not assess variation related to breed, weight, conformation, day, or time of day. We used an advanced ultrasound machine with 2 frequency ranges of phased array probes and performed measurements with concomitant ECG, ensuring that all measurements were performed with appropriate timing, with dogs placed in lateral recumbency on a portable echocardiography table, all of which may not represent routine ECC practice. This feature may have artificially increased the accuracy of the results. The goal of our study was to identify parameters to recommend for further study, and performing measurements in this way was felt to allow us to achieve this goal. In addition, it is common practice in our hospital to use phased array probes, an ECG and a portable echocardiography table. However, whereas some dogs (e.g., those in hemodynamic collapse) typically are presented in lateral recumbency and easy to transfer to an echocardiography table, patients in respiratory distress are more safely evaluated in sternal positioning. One FCU study has investigated left atrial measurements in cats performed in sternal vs lateral recumbency and found no differences. That study of cats used only one non-cardiologist compared with themself.^32^ We did not measure how long performing the FCU examination took, which would be interesting to know for application in critical patients. Four dogs with mild cardiac-related disease were included in the measurement repeatability analysis. It is possible clinicians recognized these cases, but we feel it would be unlikely they recalled specific measurements after ≥ 4 weeks had elapsed. Measuring EPSS in B-mode is influenced by the frame rates used, which may have introduced variability into the measurements, but we do not feel this factor alone is sufficient to explain the high variation and low reliability found with B-mode EPSS. Finally, we used a single cardiologist as a gold standard. We have seen that results can vary between cardiologists, and using this standard may not have been ideal.

In conclusion, 2 non-cardiologist ECC clinicians were able to obtain and measure LVEDD, a marker of LV preload, in 2 modes and 2 axes. Parasternal short-axis LVEDD-B demonstrated the lowest variation and the highest reliability, but all LVEDD parameters warrant further study. For markers of systolic function, LVESD showed the most potential to be explored further in FCU in dogs. Given the overall poor repeatability and reliability of FS and EPSS, we suggest these parameters be used cautiously, if at all, by non-cardiologist sonographers. Our study was intended to identify parameters warranting further investigation in FCU, and the results cannot be generally applied to the entire population of dogs undergoing cardiac ultrasound examination. Future studies should investigate FCU parameters performed by multiple non-cardiologists, ideally at multiple centers, using samples of dogs that reflect the clinical population undergoing FCU.

## Supplementary Material

ARTICLE_1_Appendicies_GLMM_data_aalaf090

## Off-label antimicrobial declaration

The authors declare no off-label use of antimicrobials.

## Institutional animal care and use committee or other approval declaration

Approved by Liège University (#2405, December 20, 2021).

## Abbreviations

CV: coefficient of variation
ECC: emergency and critical care
EPSS: E-point septal separation
FCU: focused cardiac ultrasound
FS: fractional shortening
GLMM: general linear mixed model
ICC: intraclass correlation coefficient
LV: left ventricle
LVEDD: left ventricular end-diastolic volume
LVEF: left ventricular ejection fraction
LVESD: left ventricular end-systolic volume
PLAX: parasternal long-axis
PSAX: parasternal short-axis
Suffix “-M”: M-mode echocardiography
Suffix “-B”: B-mode echocardiography
