# Correction to: Repeatability and reliability of left ventricular focused cardiac ultrasound parameters in dogs obtained and measured by two non-cardiologist clinicians

**DOI:** 10.1093/jvimsj/aalag059

**Published:** 2026-03-11

This is a correction to: Christopher R Kennedy, Aurélie Jourdan, Kris Gommeren, Anne-Christine Merveille, Repeatability and reliability of left ventricular focused cardiac ultrasound parameters in dogs obtained and measured by two non-cardiologist clinicians, *Journal of Veterinary Internal Medicine*, Volume 40, Issue 1, January-February 2026, aalaf090, https://doi.org/10.1093/jvimsj/aalaf090

The name of the second author has been corrected in the article to read: “Aurélie Jourdan”.

