# Supplementary material for: Repeatability and reliability of left ventricular focused cardiac ultrasound parameters in dogs obtained and measured by two non-cardiologist clinicians
Source: J Vet Intern Med. 2026 Feb 14;40(1):aalaf090. doi: 10.1093/jvimsj/aalaf090 (PMC12906273; doi:10.1093/jvimsj/aalaf090)
Supplement: ARTICLE_1_Appendicies_GLMM_data_aalaf090 [file article_1_appendicies_glmm_data_aalaf090.docx]

## GLMM models

| Parameter | Parameter estimate | SE | P-value |
| --- | --- | --- | --- |
| PLAXLVIDd_2d |  |  |  |
| Intercept | 33.1753 | 1.7101 | <.0001 |
| Mes 1 vs. Mes 2 | 0.2006 | 0.2320 | 0.3944 |
| Imager 1 vs. Imager 2 | -0.02272 | 0.2320 | 0.9227 |
| PLAXLVIDs_2d |  |  |  |
| Intercept | 23.1670 | 1.4150 | <.0001 |
| Mes 1 vs. Mes 2 | -0.3229 | 0.3954 | 0.4211 |
| Imager 1 vs. Imager 2 | 2.0446 | 0.3954 | <.0001 |
| PLAXFS_2d |  |  |  |
| Intercept | 0.3081 | 0.01425 | <.0001 |
| Mes 1 vs. Mes 2 | 0.01708 | 0.01264 | 0.1876 |
| Imager 1 vs. Imager 2 | -0.06962 | 0.01264 | <.0001 |
| PLAXLVIDd_TM |  |  |  |
| Intercept | 31.912 | 1.7216 | <.0001 |
| Mes 1 vs. Mes 2 | 0.4567 | 0.2787 | 0.1125 |
| Imager 1 vs. Imager 2 | -0.09383 | 0.2787 | 0.7389 |
| PLAXLVIDs_TM |  |  |  |
| Intercept | 20.065 | 1.3638 | <.0001 |
| Mes 1 vs. Mes 2 | 0.3293 | 0.2829 | 0.2543 |
| Imager 1 vs. Imager 2 | 1.6713 | 0.2829 | <.0001 |
| PLAXFS_TM |  |  |  |
| Intercept | 0.3853 | 0.01521 | <.0001 |
| Mes 1 vs. Mes 2 | -0.00515 | 0.01055 | 0.6292 |
| Imager 1 vs. Imager 2 | -0.06111 | 0.01055 | <.0001 |
| PLAXEPSS_2d |  |  |  |
| Intercept | 2.2287 | 0.3591 | <.0001 |
| Mes 1 vs. Mes 2 | -0.2035 | 0.1877 | 0.2876 |
| Imager 1 vs. Imager 2 | 0.08325 | 0.1877 | 0.6608 |
| PLAXEPSS_TM |  |  |  |
| Intercept | 1.1138 | 0.2013 | <.0001 |
| Mes 1 vs. Mes 2 | -0.02483 | 0.1117 | 0.8258 |
| Imager 1 vs. Imager 2 | 0.9048 | 0.1117 | <.0001 |
| PSAXLVIDd_2d |  |  |  |
| Intercept | 34.134 | 1.7464 | <.0001 |
| Mes 1 vs. Mes 2 | -0.2185 | 0.2647 | 0.4162 |
| Imager 1 vs. Imager 2 | -1.5032 | 0.2647 | <.0001 |
| PSAXLVIDs_2d |  |  |  |
| Intercept | 23.4267 | 1.4687 | <.0001 |
| Mes 1 vs. Mes 2 | -0.04953 | 0.2757 | 0.8587 |
| Imager 1 vs. Imager 2 | -0.1028 | 0.2757 | 0.7121 |
| PSAXFS_2d |  |  |  |
| Intercept | 0.3247 | 0.01499 | <.0001 |
| Mes 1 vs. Mes 2 | -0.00361 | 0.009397 | 0.7037 |
| Imager 1 vs. Imager 2 | -0.03143 | 0.009397 | 0.0024 |
| PSAXLVIDd_TM |  |  |  |
| Intercept | 33.1059 | 1.7454 | <.0001 |
| Mes 1 vs. Mes 2 | 0.2082 | 0.2258 | 0.3643 |
| Imager 1 vs. Imager 2 | -0.1245 | 0.2258 | 0.5857 |
| PSAXLVIDs_TM |  |  |  |
| Intercept | 21.002 | 1.3951 | <.0001 |
| Mes 1 vs. Mes 2 | 0.03993 | 0.2531 | 0.8758 |
| Imager 1 vs. Imager 2 | 1.5211 | 0.2531 | <.0001 |
| PSAXFS_TM |  |  |  |
| Intercept | 0.3757 | 0.0141 | <.0001 |
| Mes 1 vs. Mes 2 | 0.002531 | 0.007778 | 0.7472 |
| Imager 1 vs. Imager 2 | -0.05199 | 0.007778 | <.0001 |
| PSAXEPSS_2d |  |  |  |
| Intercept | 2.9328 | 0.3427 | <.0001 |
| Mes 1 vs. Mes 2 | -0.108 | 0.1764 | 0.5453 |
| Imager 1 vs. Imager 2 | -0.2297 | 0.1764 | 0.2034 |
| PSAXEPSS_TM |  |  |  |
| Intercept | 1.8516 | 0.3086 | <.0001 |
| Mes 1 vs. Mes 2 | -0.08397 | 0.1125 | 0.4616 |
| Imager 1 vs. Imager 2 | 0.6647 | 0.1125 | <.0001 |

| nomvari | Frequency | Percent | Cumulative Frequency | Cumulative Percent |
| --- | --- | --- | --- | --- |
| PLAX_FS_2d | 58 | 100.00 | 58 | 100.00 |

Comparison Mes 1 vs. Mes 2

| Model Information | |
| --- | --- |
| Data Set | WORK.KENN |
| Dependent Variable | valeur |
| Covariance Structure | Compound Symmetry |
| Subject Effect | numdog |
| Estimation Method | REML |
| Residual Variance Method | Profile |
| Fixed Effects SE Method | Model-Based |
| Degrees of Freedom Method | Between-Within |

| Effect | numimager | MES | Estimate | Standard Error | DF | t Value | Pr > \|t\| |
| --- | --- | --- | --- | --- | --- | --- | --- |
| Intercept |  |  | 0.3081 | 0.01425 | 28 | 21.62 | <.0001 |
| MES |  | 1 | 0.01708 | 0.01264 | 28 | 1.35 | 0.1876 |
| MES |  | 2 | 0 | . | . | . | . |
| numimager | 1 |  | -0.06962 | 0.01264 | 28 | -5.51 | <.0001 |
| numimager | 2 |  | 0 | . | . | . | . |

| Type 3 Tests of Fixed Effects | | | | |
| --- | --- | --- | --- | --- |
| Effect | Num DF | Den DF | F Value | Pr > F |
| MES | 1 | 28 | 1.82 | 0.1876 |
| numimager | 1 | 28 | 30.32 | <.0001 |

Comparison Mes 1 vs. Mes 2

| nomvari | Frequency | Percent | Cumulative Frequency | Cumulative Percent |
| --- | --- | --- | --- | --- |
| PLAX_LVIDd_TM | 58 | 100.00 | 58 | 100.00 |

Comparison Mes 1 vs. Mes 2

| Model Information | |
| --- | --- |
| Data Set | WORK.KENN |
| Dependent Variable | valeur |
| Covariance Structure | Compound Symmetry |
| Subject Effect | numdog |
| Estimation Method | REML |
| Residual Variance Method | Profile |
| Fixed Effects SE Method | Model-Based |
| Degrees of Freedom Method | Between-Within |

| Solution for Fixed Effects | | | | | | | |
| --- | --- | --- | --- | --- | --- | --- | --- |
| Effect | numimager | MES | Estimate | Standard Error | DF | t Value | Pr > \|t\| |
| Intercept |  |  | 31.9120 | 1.7216 | 28 | 18.54 | <.0001 |
| MES |  | 1 | 0.4567 | 0.2787 | 28 | 1.64 | 0.1125 |
| MES |  | 2 | 0 | . | . | . | . |
| numimager | 1 |  | -0.09383 | 0.2787 | 28 | -0.34 | 0.7389 |
| numimager | 2 |  | 0 | . | . | . | . |

| Type 3 Tests of Fixed Effects | | | | |
| --- | --- | --- | --- | --- |
| Effect | Num DF | Den DF | F Value | Pr > F |
| MES | 1 | 28 | 2.69 | 0.1125 |
| numimager | 1 | 28 | 0.11 | 0.7389 |

Comparison Mes 1 vs. Mes 2

| nomvari | Frequency | Percent | Cumulative Frequency | Cumulative Percent |
| --- | --- | --- | --- | --- |
| PLAX_LVIDs_TM | 58 | 100.00 | 58 | 100.00 |

Comparison Mes 1 vs. Mes 2

| Model Information | |
| --- | --- |
| Data Set | WORK.KENN |
| Dependent Variable | valeur |
| Covariance Structure | Compound Symmetry |
| Subject Effect | numdog |
| Estimation Method | REML |
| Residual Variance Method | Profile |
| Fixed Effects SE Method | Model-Based |
| Degrees of Freedom Method | Between-Within |

| Solution for Fixed Effects | | | | | | | |
| --- | --- | --- | --- | --- | --- | --- | --- |
| Effect | numimager | MES | Estimate | Standard Error | DF | t Value | Pr > \|t\| |
| Intercept |  |  | 20.0650 | 1.3638 | 28 | 14.71 | <.0001 |
| MES |  | 1 | 0.3293 | 0.2829 | 28 | 1.16 | 0.2543 |
| MES |  | 2 | 0 | . | . | . | . |
| numimager | 1 |  | 1.6713 | 0.2829 | 28 | 5.91 | <.0001 |
| numimager | 2 |  | 0 | . | . | . | . |

| Type 3 Tests of Fixed Effects | | | | |
| --- | --- | --- | --- | --- |
| Effect | Num DF | Den DF | F Value | Pr > F |
| MES | 1 | 28 | 1.35 | 0.2543 |
| numimager | 1 | 28 | 34.89 | <.0001 |

Comparison Mes 1 vs. Mes 2

| nomvari | Frequency | Percent | Cumulative Frequency | Cumulative Percent |
| --- | --- | --- | --- | --- |
| PLAX_FS_TM | 58 | 100.00 | 58 | 100.00 |

Comparison Mes 1 vs. Mes 2

| Model Information | |
| --- | --- |
| Data Set | WORK.KENN |
| Dependent Variable | valeur |
| Covariance Structure | Compound Symmetry |
| Subject Effect | numdog |
| Estimation Method | REML |
| Residual Variance Method | Profile |
| Fixed Effects SE Method | Model-Based |
| Degrees of Freedom Method | Between-Within |

| Solution for Fixed Effects | | | | | | | |
| --- | --- | --- | --- | --- | --- | --- | --- |
| Effect | numimager | MES | Estimate | Standard Error | DF | t Value | Pr > \|t\| |
| Intercept |  |  | 0.3853 | 0.01521 | 28 | 25.34 | <.0001 |
| MES |  | 1 | -0.00515 | 0.01055 | 28 | -0.49 | 0.6292 |
| MES |  | 2 | 0 | . | . | . | . |
| numimager | 1 |  | -0.06111 | 0.01055 | 28 | -5.79 | <.0001 |
| numimager | 2 |  | 0 | . | . | . | . |

| Type 3 Tests of Fixed Effects | | | | |
| --- | --- | --- | --- | --- |
| Effect | Num DF | Den DF | F Value | Pr > F |
| MES | 1 | 28 | 0.24 | 0.6292 |
| numimager | 1 | 28 | 33.54 | <.0001 |

Comparison Mes 1 vs. Mes 2

| nomvari | Frequency | Percent | Cumulative Frequency | Cumulative Percent |
| --- | --- | --- | --- | --- |
| PLAX_EPSS_2d | 58 | 100.00 | 58 | 100.00 |

Comparison Mes 1 vs. Mes 2

| Model Information | |
| --- | --- |
| Data Set | WORK.KENN |
| Dependent Variable | valeur |
| Covariance Structure | Compound Symmetry |
| Subject Effect | numdog |
| Estimation Method | REML |
| Residual Variance Method | Profile |
| Fixed Effects SE Method | Model-Based |
| Degrees of Freedom Method | Between-Within |

| Solution for Fixed Effects | | | | | | | |
| --- | --- | --- | --- | --- | --- | --- | --- |
| Effect | numimager | MES | Estimate | Standard Error | DF | t Value | Pr > \|t\| |
| Intercept |  |  | 2.2287 | 0.3591 | 28 | 6.21 | <.0001 |
| MES |  | 1 | -0.2035 | 0.1877 | 28 | -1.08 | 0.2876 |
| MES |  | 2 | 0 | . | . | . | . |
| numimager | 1 |  | 0.08325 | 0.1877 | 28 | 0.44 | 0.6608 |
| numimager | 2 |  | 0 | . | . | . | . |

| Type 3 Tests of Fixed Effects | | | | |
| --- | --- | --- | --- | --- |
| Effect | Num DF | Den DF | F Value | Pr > F |
| MES | 1 | 28 | 1.18 | 0.2876 |
| numimager | 1 | 28 | 0.20 | 0.6608 |

Comparison Mes 1 vs. Mes 2

| nomvari | Frequency | Percent | Cumulative Frequency | Cumulative Percent |
| --- | --- | --- | --- | --- |
| PLAX_EPSS_TM | 58 | 100.00 | 58 | 100.00 |

Comparison Mes 1 vs. Mes 2

| Model Information | |
| --- | --- |
| Data Set | WORK.KENN |
| Dependent Variable | valeur |
| Covariance Structure | Compound Symmetry |
| Subject Effect | numdog |
| Estimation Method | REML |
| Residual Variance Method | Profile |
| Fixed Effects SE Method | Model-Based |
| Degrees of Freedom Method | Between-Within |

| Solution for Fixed Effects | | | | | | | |
| --- | --- | --- | --- | --- | --- | --- | --- |
| Effect | numimager | MES | Estimate | Standard Error | DF | t Value | Pr > \|t\| |
| Intercept |  |  | 1.1138 | 0.2013 | 28 | 5.53 | <.0001 |
| MES |  | 1 | -0.02483 | 0.1117 | 28 | -0.22 | 0.8258 |
| MES |  | 2 | 0 | . | . | . | . |
| numimager | 1 |  | 0.9048 | 0.1117 | 28 | 8.10 | <.0001 |
| numimager | 2 |  | 0 | . | . | . | . |

| Type 3 Tests of Fixed Effects | | | | |
| --- | --- | --- | --- | --- |
| Effect | Num DF | Den DF | F Value | Pr > F |
| MES | 1 | 28 | 0.05 | 0.8258 |
| numimager | 1 | 28 | 65.57 | <.0001 |

Comparison Mes 1 vs. Mes 2

| nomvari | Frequency | Percent | Cumulative Frequency | Cumulative Percent |
| --- | --- | --- | --- | --- |
| PSAX_LVIDd_2d | 58 | 100.00 | 58 | 100.00 |

Comparison Mes 1 vs. Mes 2

| Model Information | |
| --- | --- |
| Data Set | WORK.KENN |
| Dependent Variable | valeur |
| Covariance Structure | Compound Symmetry |
| Subject Effect | numdog |
| Estimation Method | REML |
| Residual Variance Method | Profile |
| Fixed Effects SE Method | Model-Based |
| Degrees of Freedom Method | Between-Within |

| Solution for Fixed Effects | | | | | | | |
| --- | --- | --- | --- | --- | --- | --- | --- |
| Effect | numimager | MES | Estimate | Standard Error | DF | t Value | Pr > \|t\| |
| Intercept |  |  | 34.1340 | 1.7464 | 28 | 19.55 | <.0001 |
| MES |  | 1 | -0.2185 | 0.2647 | 28 | -0.83 | 0.4162 |
| MES |  | 2 | 0 | . | . | . | . |
| numimager | 1 |  | -1.5032 | 0.2647 | 28 | -5.68 | <.0001 |
| numimager | 2 |  | 0 | . | . | . | . |

| Type 3 Tests of Fixed Effects | | | | |
| --- | --- | --- | --- | --- |
| Effect | Num DF | Den DF | F Value | Pr > F |
| MES | 1 | 28 | 0.68 | 0.4162 |
| numimager | 1 | 28 | 32.25 | <.0001 |

Comparison Mes 1 vs. Mes 2

| nomvari | Frequency | Percent | Cumulative Frequency | Cumulative Percent |
| --- | --- | --- | --- | --- |
| PSAX_LVIDs_2d | 58 | 100.00 | 58 | 100.00 |

Comparison Mes 1 vs. Mes 2

| Model Information | |
| --- | --- |
| Data Set | WORK.KENN |
| Dependent Variable | valeur |
| Covariance Structure | Compound Symmetry |
| Subject Effect | numdog |
| Estimation Method | REML |
| Residual Variance Method | Profile |
| Fixed Effects SE Method | Model-Based |
| Degrees of Freedom Method | Between-Within |

| Solution for Fixed Effects | | | | | | | |
| --- | --- | --- | --- | --- | --- | --- | --- |
| Effect | numimager | MES | Estimate | Standard Error | DF | t Value | Pr > \|t\| |
| Intercept |  |  | 23.4267 | 1.4687 | 28 | 15.95 | <.0001 |
| MES |  | 1 | -0.04953 | 0.2757 | 28 | -0.18 | 0.8587 |
| MES |  | 2 | 0 | . | . | . | . |
| numimager | 1 |  | -0.1028 | 0.2757 | 28 | -0.37 | 0.7121 |
| numimager | 2 |  | 0 | . | . | . | . |

| Type 3 Tests of Fixed Effects | | | | |
| --- | --- | --- | --- | --- |
| Effect | Num DF | Den DF | F Value | Pr > F |
| MES | 1 | 28 | 0.03 | 0.8587 |
| numimager | 1 | 28 | 0.14 | 0.7121 |

Comparison Mes 1 vs. Mes 2

| nomvari | Frequency | Percent | Cumulative Frequency | Cumulative Percent |
| --- | --- | --- | --- | --- |
| PSAX_FS_2d | 58 | 100.00 | 58 | 100.00 |

Comparison Mes 1 vs. Mes 2

| Model Information | |
| --- | --- |
| Data Set | WORK.KENN |
| Dependent Variable | valeur |
| Covariance Structure | Compound Symmetry |
| Subject Effect | numdog |
| Estimation Method | REML |
| Residual Variance Method | Profile |
| Fixed Effects SE Method | Model-Based |
| Degrees of Freedom Method | Between-Within |

| Solution for Fixed Effects | | | | | | | |
| --- | --- | --- | --- | --- | --- | --- | --- |
| Effect | numimager | MES | Estimate | Standard Error | DF | t Value | Pr > \|t\| |
| Intercept |  |  | 0.3247 | 0.01499 | 28 | 21.66 | <.0001 |
| MES |  | 1 | -0.00361 | 0.009397 | 28 | -0.38 | 0.7037 |
| MES |  | 2 | 0 | . | . | . | . |
| numimager | 1 |  | -0.03143 | 0.009397 | 28 | -3.34 | 0.0024 |
| numimager | 2 |  | 0 | . | . | . | . |

| Type 3 Tests of Fixed Effects | | | | |
| --- | --- | --- | --- | --- |
| Effect | Num DF | Den DF | F Value | Pr > F |
| MES | 1 | 28 | 0.15 | 0.7037 |
| numimager | 1 | 28 | 11.19 | 0.0024 |

Comparison Mes 1 vs. Mes 2

| nomvari | Frequency | Percent | Cumulative Frequency | Cumulative Percent |
| --- | --- | --- | --- | --- |
| PSAX_LVIDd_TM | 58 | 100.00 | 58 | 100.00 |

Comparison Mes 1 vs. Mes 2

| Model Information | |
| --- | --- |
| Data Set | WORK.KENN |
| Dependent Variable | valeur |
| Covariance Structure | Compound Symmetry |
| Subject Effect | numdog |
| Estimation Method | REML |
| Residual Variance Method | Profile |
| Fixed Effects SE Method | Model-Based |
| Degrees of Freedom Method | Between-Within |

| Solution for Fixed Effects | | | | | | | |
| --- | --- | --- | --- | --- | --- | --- | --- |
| Effect | numimager | MES | Estimate | Standard Error | DF | t Value | Pr > \|t\| |
| Intercept |  |  | 33.1059 | 1.7454 | 28 | 18.97 | <.0001 |
| MES |  | 1 | 0.2082 | 0.2258 | 28 | 0.92 | 0.3643 |
| MES |  | 2 | 0 | . | . | . | . |
| numimager | 1 |  | -0.1245 | 0.2258 | 28 | -0.55 | 0.5857 |
| numimager | 2 |  | 0 | . | . | . | . |

| Type 3 Tests of Fixed Effects | | | | |
| --- | --- | --- | --- | --- |
| Effect | Num DF | Den DF | F Value | Pr > F |
| MES | 1 | 28 | 0.85 | 0.3643 |
| numimager | 1 | 28 | 0.30 | 0.5857 |

Comparison Mes 1 vs. Mes 2

| nomvari | Frequency | Percent | Cumulative Frequency | Cumulative Percent |
| --- | --- | --- | --- | --- |
| PSAX_LVIDs_TM | 58 | 100.00 | 58 | 100.00 |

Comparison Mes 1 vs. Mes 2

| Model Information | |
| --- | --- |
| Data Set | WORK.KENN |
| Dependent Variable | valeur |
| Covariance Structure | Compound Symmetry |
| Subject Effect | numdog |
| Estimation Method | REML |
| Residual Variance Method | Profile |
| Fixed Effects SE Method | Model-Based |
| Degrees of Freedom Method | Between-Within |

| Solution for Fixed Effects | | | | | | | |
| --- | --- | --- | --- | --- | --- | --- | --- |
| Effect | numimager | MES | Estimate | Standard Error | DF | t Value | Pr > \|t\| |
| Intercept |  |  | 21.0020 | 1.3951 | 28 | 15.05 | <.0001 |
| MES |  | 1 | 0.03993 | 0.2531 | 28 | 0.16 | 0.8758 |
| MES |  | 2 | 0 | . | . | . | . |
| numimager | 1 |  | 1.5211 | 0.2531 | 28 | 6.01 | <.0001 |
| numimager | 2 |  | 0 | . | . | . | . |

| Type 3 Tests of Fixed Effects | | | | |
| --- | --- | --- | --- | --- |
| Effect | Num DF | Den DF | F Value | Pr > F |
| MES | 1 | 28 | 0.02 | 0.8758 |
| numimager | 1 | 28 | 36.11 | <.0001 |

Comparison Mes 1 vs. Mes 2

| nomvari | Frequency | Percent | Cumulative Frequency | Cumulative Percent |
| --- | --- | --- | --- | --- |
| PSAX_FS_TM | 58 | 100.00 | 58 | 100.00 |

Comparison Mes 1 vs. Mes 2

| Model Information | |
| --- | --- |
| Data Set | WORK.KENN |
| Dependent Variable | valeur |
| Covariance Structure | Compound Symmetry |
| Subject Effect | numdog |
| Estimation Method | REML |
| Residual Variance Method | Profile |
| Fixed Effects SE Method | Model-Based |
| Degrees of Freedom Method | Between-Within |

| Solution for Fixed Effects | | | | | | | |
| --- | --- | --- | --- | --- | --- | --- | --- |
| Effect | numimager | MES | Estimate | Standard Error | DF | t Value | Pr > \|t\| |
| Intercept |  |  | 0.3757 | 0.01410 | 28 | 26.65 | <.0001 |
| MES |  | 1 | 0.002531 | 0.007778 | 28 | 0.33 | 0.7472 |
| MES |  | 2 | 0 | . | . | . | . |
| numimager | 1 |  | -0.05199 | 0.007778 | 28 | -6.68 | <.0001 |
| numimager | 2 |  | 0 | . | . | . | . |

| Type 3 Tests of Fixed Effects | | | | |
| --- | --- | --- | --- | --- |
| Effect | Num DF | Den DF | F Value | Pr > F |
| MES | 1 | 28 | 0.11 | 0.7472 |
| numimager | 1 | 28 | 44.69 | <.0001 |

Comparison Mes 1 vs. Mes 2

| nomvari | Frequency | Percent | Cumulative Frequency | Cumulative Percent |
| --- | --- | --- | --- | --- |
| PSAX_EPSS_2d | 58 | 100.00 | 58 | 100.00 |

Comparison Mes 1 vs. Mes 2

| Model Information | |
| --- | --- |
| Data Set | WORK.KENN |
| Dependent Variable | valeur |
| Covariance Structure | Compound Symmetry |
| Subject Effect | numdog |
| Estimation Method | REML |
| Residual Variance Method | Profile |
| Fixed Effects SE Method | Model-Based |
| Degrees of Freedom Method | Between-Within |

| Solution for Fixed Effects | | | | | | | |
| --- | --- | --- | --- | --- | --- | --- | --- |
| Effect | numimager | MES | Estimate | Standard Error | DF | t Value | Pr > \|t\| |
| Intercept |  |  | 2.9328 | 0.3427 | 28 | 8.56 | <.0001 |
| MES |  | 1 | -0.1080 | 0.1764 | 28 | -0.61 | 0.5453 |
| MES |  | 2 | 0 | . | . | . | . |
| numimager | 1 |  | -0.2297 | 0.1764 | 28 | -1.30 | 0.2034 |
| numimager | 2 |  | 0 | . | . | . | . |

| Type 3 Tests of Fixed Effects | | | | |
| --- | --- | --- | --- | --- |
| Effect | Num DF | Den DF | F Value | Pr > F |
| MES | 1 | 28 | 0.37 | 0.5453 |
| numimager | 1 | 28 | 1.70 | 0.2034 |

Comparison Mes 1 vs. Mes 2

| nomvari | Frequency | Percent | Cumulative Frequency | Cumulative Percent |
| --- | --- | --- | --- | --- |
| PSAX_EPSS_TM | 58 | 100.00 | 58 | 100.00 |

Comparison Mes 1 vs. Mes 2

| Model Information | |
| --- | --- |
| Data Set | WORK.KENN |
| Dependent Variable | valeur |
| Covariance Structure | Compound Symmetry |
| Subject Effect | numdog |
| Estimation Method | REML |
| Residual Variance Method | Profile |
| Fixed Effects SE Method | Model-Based |
| Degrees of Freedom Method | Between-Within |

| Solution for Fixed Effects | | | | | | | |
| --- | --- | --- | --- | --- | --- | --- | --- |
| Effect | numimager | MES | Estimate | Standard Error | DF | t Value | Pr > \|t\| |
| Intercept |  |  | 1.8516 | 0.3086 | 28 | 6.00 | <.0001 |
| MES |  | 1 | -0.08397 | 0.1125 | 28 | -0.75 | 0.4616 |
| MES |  | 2 | 0 | . | . | . | . |
| numimager | 1 |  | 0.6647 | 0.1125 | 28 | 5.91 | <.0001 |
| numimager | 2 |  | 0 | . | . | . | . |

| Type 3 Tests of Fixed Effects | | | | |
| --- | --- | --- | --- | --- |
| Effect | Num DF | Den DF | F Value | Pr > F |
| MES | 1 | 28 | 0.56 | 0.4616 |
| numimager | 1 | 28 | 34.92 | <.0001 |

| Intercept | 0.3081 | 0.01425 | <.0001 |
| --- | --- | --- | --- |
| MES | 0.01708 | 0.01264 | 0.1876 |
| numimager | -0.06962 | 0.01264 | <.0001 |
|  |  |  |  |
| Intercept | 31.912 | 1.7216 | <.0001 |
| MES | 0.4567 | 0.2787 | 0.1125 |
| numimager | -0.09383 | 0.2787 | 0.7389 |
|  |  |  |  |
| Intercept | 20.065 | 1.3638 | <.0001 |
| MES | 0.3293 | 0.2829 | 0.2543 |
| numimager | 1.6713 | 0.2829 | <.0001 |
|  |  |  |  |
| Intercept | 0.3853 | 0.01521 | <.0001 |
| MES | -0.00515 | 0.01055 | 0.6292 |
| numimager | -0.06111 | 0.01055 | <.0001 |
|  |  |  |  |
| Intercept | 2.2287 | 0.3591 | <.0001 |
| MES | -0.2035 | 0.1877 | 0.2876 |
| numimager | 0.08325 | 0.1877 | 0.6608 |
|  |  |  |  |
| Intercept | 1.1138 | 0.2013 | <.0001 |
| MES | -0.02483 | 0.1117 | 0.8258 |
| numimager | 0.9048 | 0.1117 | <.0001 |
|  |  |  |  |
| Intercept | 34.134 | 1.7464 | <.0001 |
| MES | -0.2185 | 0.2647 | 0.4162 |
| numimager | -1.5032 | 0.2647 | <.0001 |
|  |  |  |  |
| Intercept | 23.4267 | 1.4687 | <.0001 |
| MES | -0.04953 | 0.2757 | 0.8587 |
| numimager | -0.1028 | 0.2757 | 0.7121 |
|  |  |  |  |
| Intercept | 0.3247 | 0.01499 | <.0001 |
| MES | -0.00361 | 0.009397 | 0.7037 |
| numimager | -0.03143 | 0.009397 | 0.0024 |
|  |  |  |  |
| Intercept | 33.1059 | 1.7454 | <.0001 |
| MES | 0.2082 | 0.2258 | 0.3643 |
| numimager | -0.1245 | 0.2258 | 0.5857 |
|  |  |  |  |
| Intercept | 21.002 | 1.3951 | <.0001 |
| MES | 0.03993 | 0.2531 | 0.8758 |
| numimager | 1.5211 | 0.2531 | <.0001 |
|  |  |  |  |
| Intercept | 0.3757 | 0.0141 | <.0001 |
| MES | 0.002531 | 0.007778 | 0.7472 |
| numimager | -0.05199 | 0.007778 | <.0001 |
